# Supplementary material for: Cost-effective DNA extraction method optimized for high yield and long fragments from coastal sediments
Source: PLoS One. 2026 Feb 24;21(2):e0343743. doi: 10.1371/journal.pone.0343743 (PMC12931794; doi:10.1371/journal.pone.0343743)
Supplement: S1 File — (DOCX) [file pone.0343743.s001.docx]

**Summary of optimization methods on sediment EstFS.**

| Method n° | Lysis buffer | CTAB formulation | CTAB %v/v | SDS %v/v | PPS | DNA Concentration (ng µL^-1^) | DNA Yield (µg_DNA_ g_dw_^-1^) |
| --- | --- | --- | --- | --- | --- | --- | --- |
| 1 | TEN | 2 | 2 | 2 | Yes | Not quantified | Not quantified |
| 2 | TSE | 1 | 2 | 1 | Yes | <0.025* | <0.008* |
| 3 | TSE | 2 | 2 | 1 | Yes | <0.025* | <0.008* |
| 4 | TSE | 1 | 1 | 1 | Yes | 0.913 | 0.269 |
| 5 | TSE | 2 | 1 | 1 | Yes | 6.123^a^ | 1.819^a^ |
| 6 | TEN | 1 | 2 | 1 | Yes | 1.221 | 0.334 |
| 7 | TEN | 2 | 2 | 1 | Yes | 0.900 | 0.265 |
| 8 | TEN | 1 | 1 | 1 | Yes | 1.285 | 0.373 |
| 9 | TSE | 1 | 1 | 1 | No | <0.025* | <0.008* |
| 10 | TSE | 1 | 1 | 2 | Yes | 0.907 | 0.279 |
| 11 | TSE | 1 | 1 | 2 | No | 1.654 | 0.514 |
| 12 | TEN | 1 | 1 | 2 | No | 1.281 | 0.379 |
| 13 | TEN | 1 | 1 | 1 | No | 1.278 | 0.380 |
| 14 | TEN | 1 | 1 | 1 | Yes | 0.888 | 0.268 |
| LM | TEN | 2 | 1 | 1 | Yes | 1.978 | 0.577 |

TEN buffer (Tris-HCl 100 mM, EDTA 10 mM, NaCl 1.5 M, Na_2_HPO_4_ 100 mM, pH 8), TSE buffer (Tris-HCl 50 mM, EDTA 20 mM, NaCl 400 mM, Sucrose 750 mm, pH 9). CTAB formulation: Hexadecyltrimethylammonium bromide (1); Cetyltrimethylammonium bromide (2). CTAB and SDS concentrations are final concentrations. DNA yield expressed in microgram of DNA per gram of sediment (dry weight, dw). * DNA concentration below detection limit. ^a^ Wood artefact in the sediment.

**Genomic DNA electrophoresis of optimization methods.**

*: Method LM. The white arrow indicates the 20 kbp band on the GeneRuler 1 kb Plus DNA Ladder (ThermoFisher). A volume of 10µl were added the well for each sample. The 0.8% TBE agarose gel was run for 30 min at 135V.
