# Supplementary figures and images for: Cost-effective DNA extraction method optimized for high yield and long fragments from coastal sediments
Source: PLoS One. 2026 Feb 24;21(2):e0343743. doi: 10.1371/journal.pone.0343743 (PMC12931794; doi:10.1371/journal.pone.0343743)

A

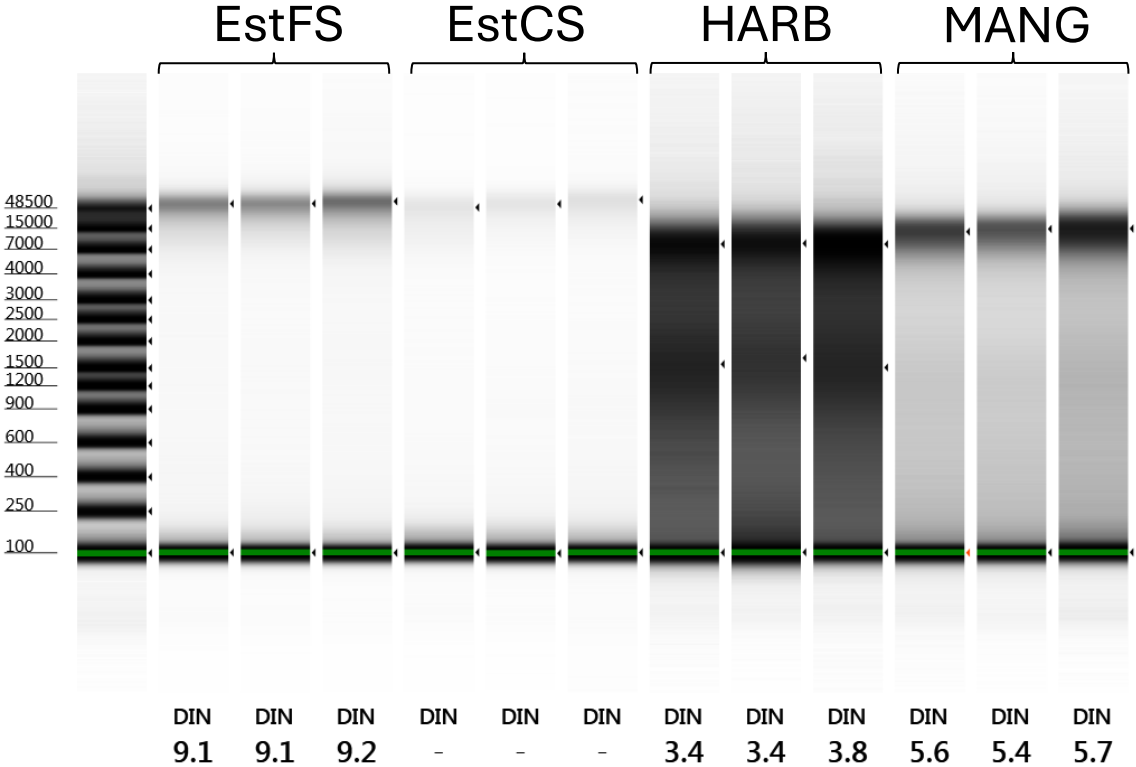

B

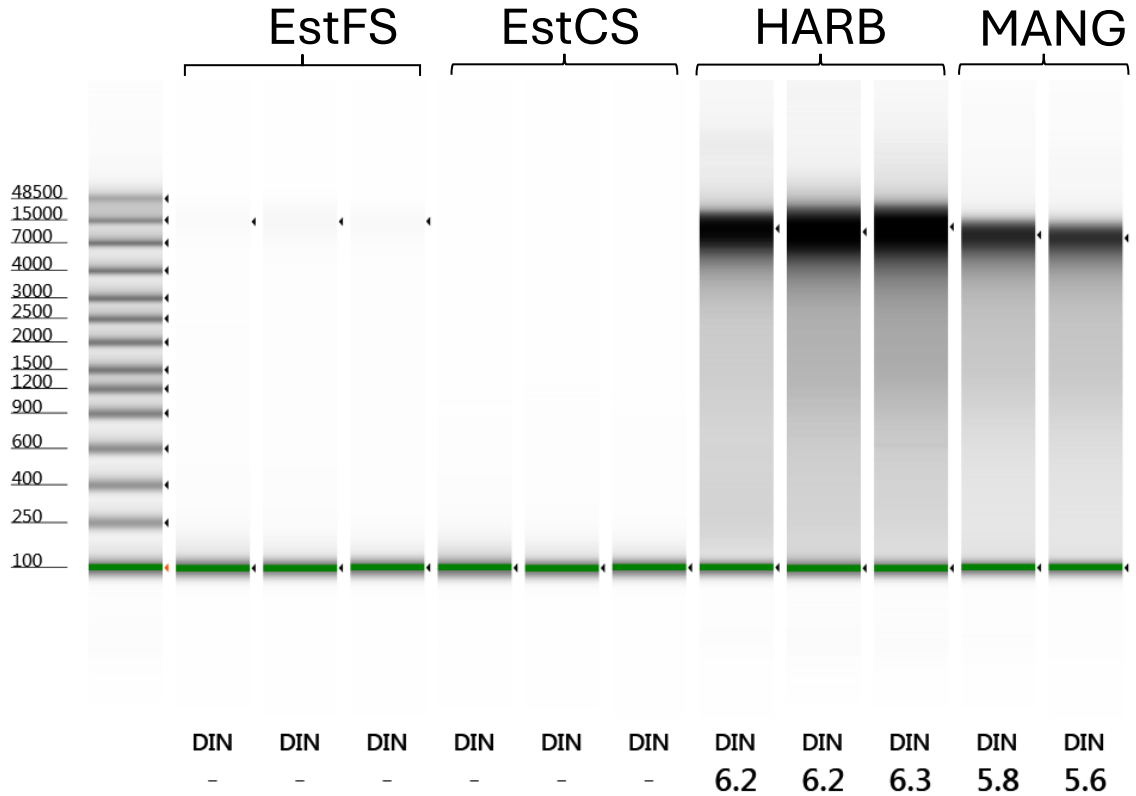

C

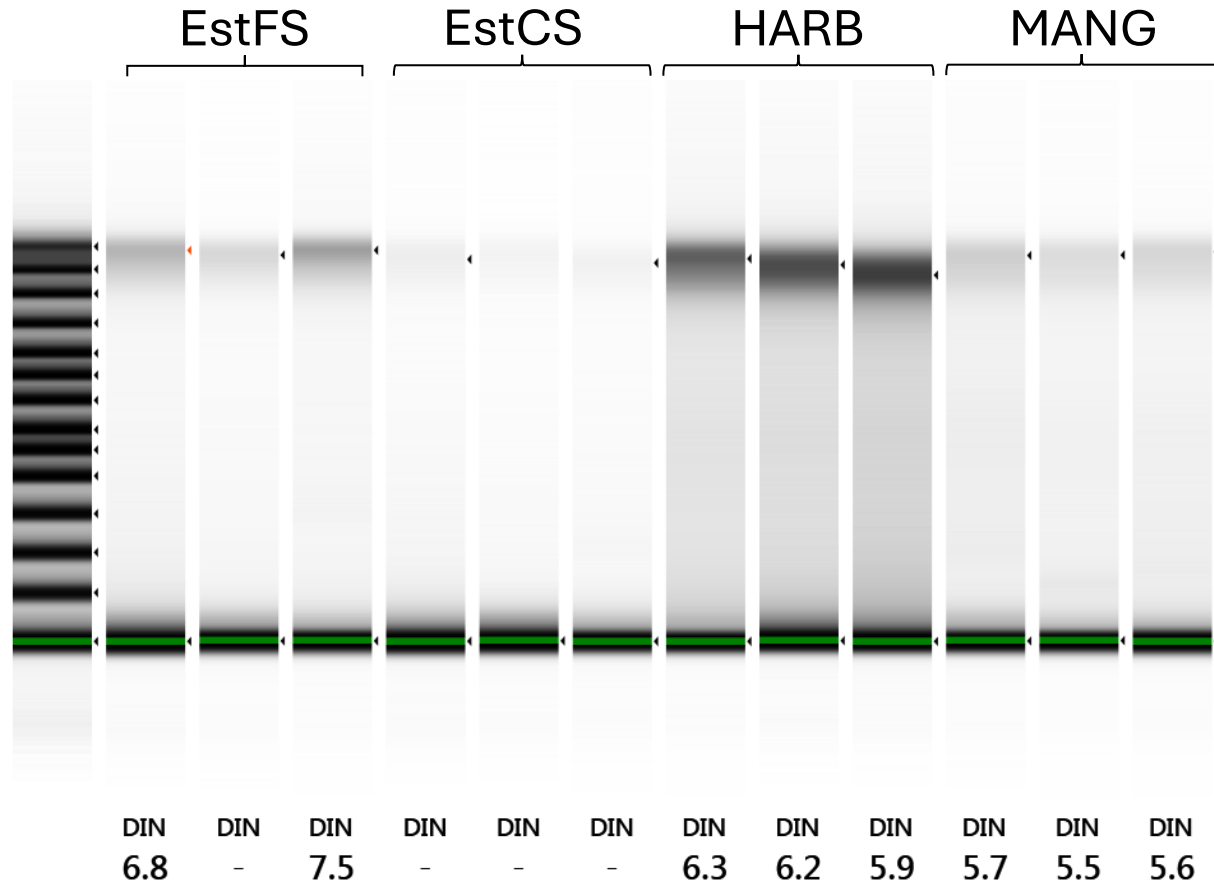

Supplement: S1 Fig — (A) Laboratory-made method (LM). (B) PowerSoil Pro (PSP). (C) PowerMax Soil (PM). (PDF) [file pone.0343743.s003.pdf]

# Prokaryotes

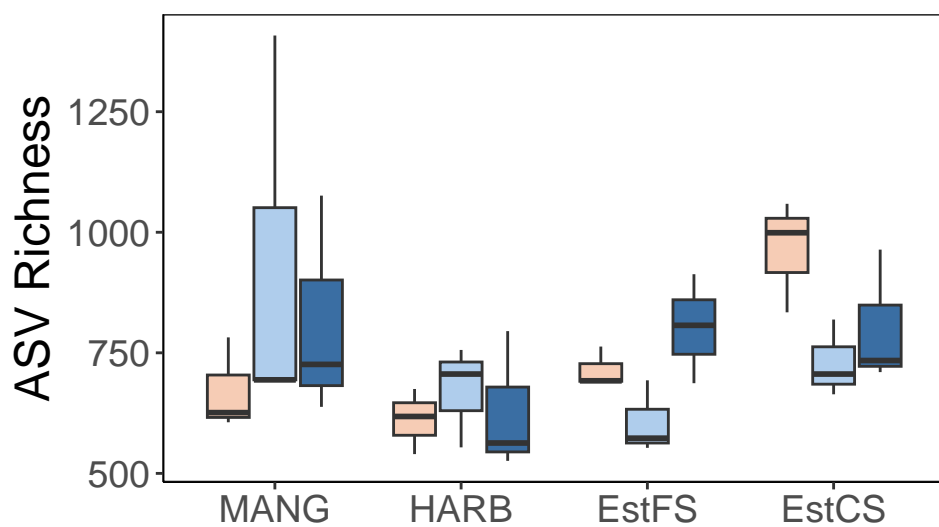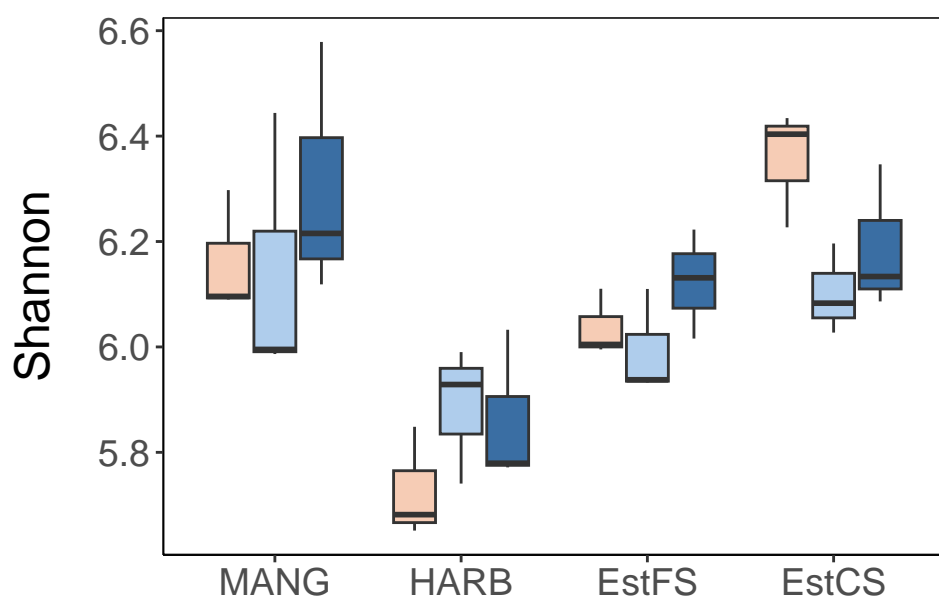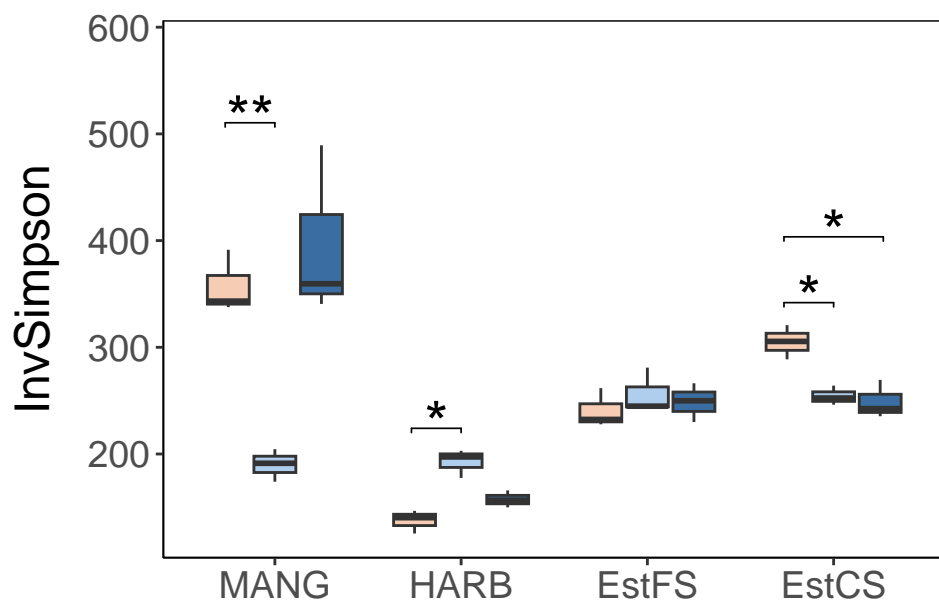

# Eukaryotes

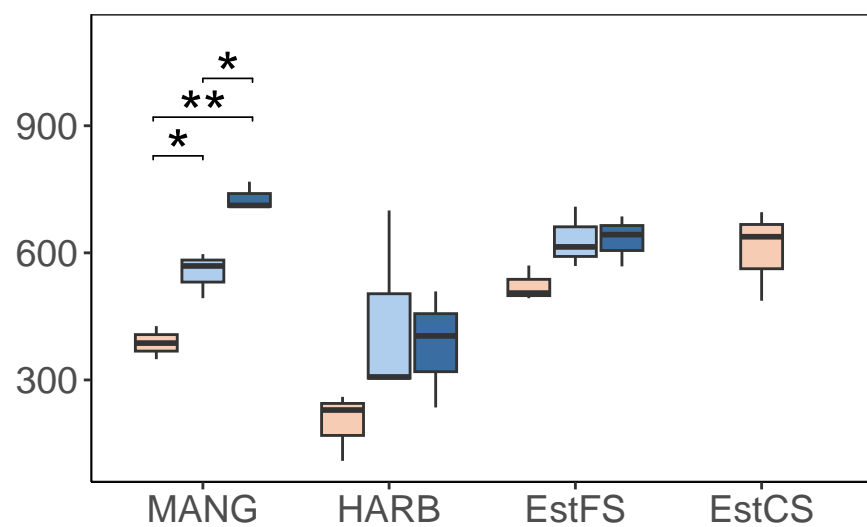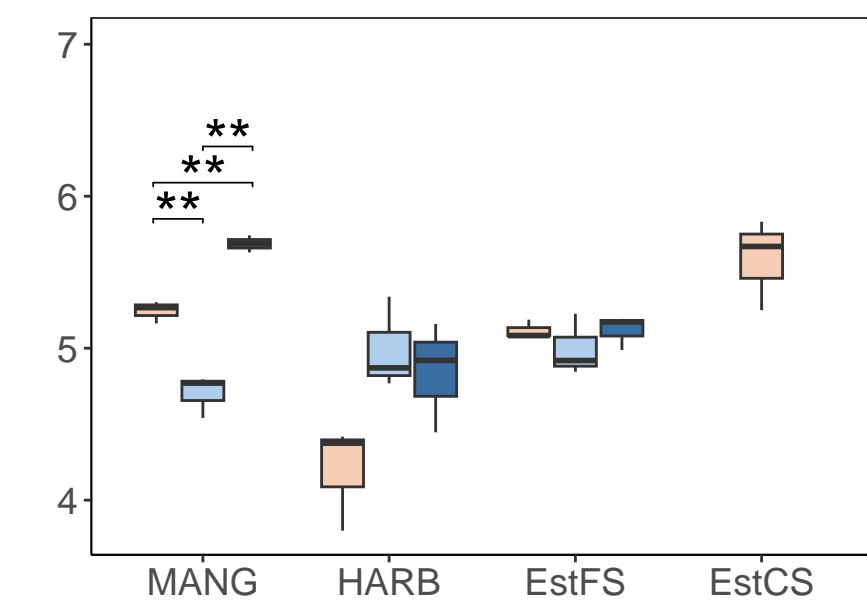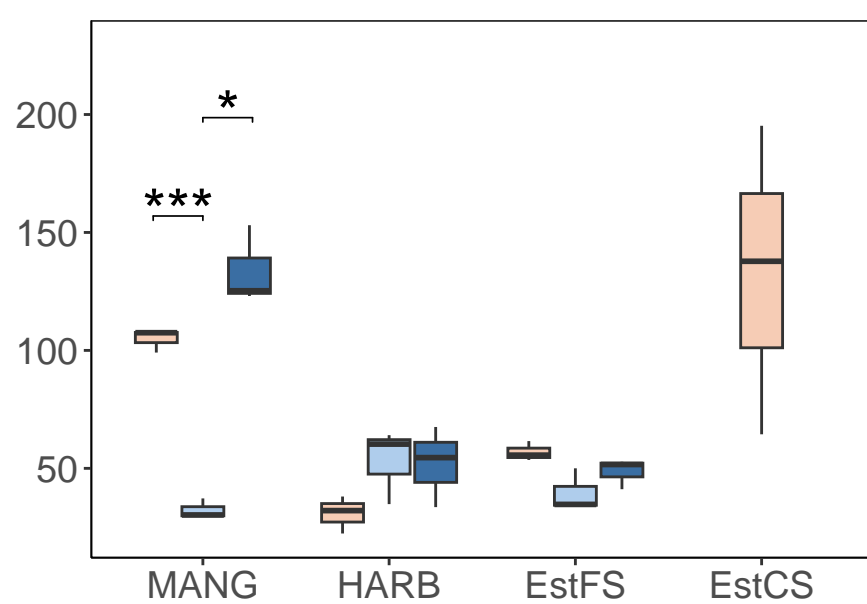

LM PSP PM

Supplement: S2 Fig — Diversity metrics include ASV richness, Shannon Index, and Inverse Simpson Index (InvSimpson). The left panel represents prokaryotic communities, while the right panel represents eukaryotic communities. Statistical significance was assessed using a t-test. LM: Laboratory-made method. PSP: PowerSoil Pro. PM: PowerMax Soil. * p < 0.05. ** p < 0.01. *** p < 0.001. (PDF) [file pone.0343743.s004.pdf]

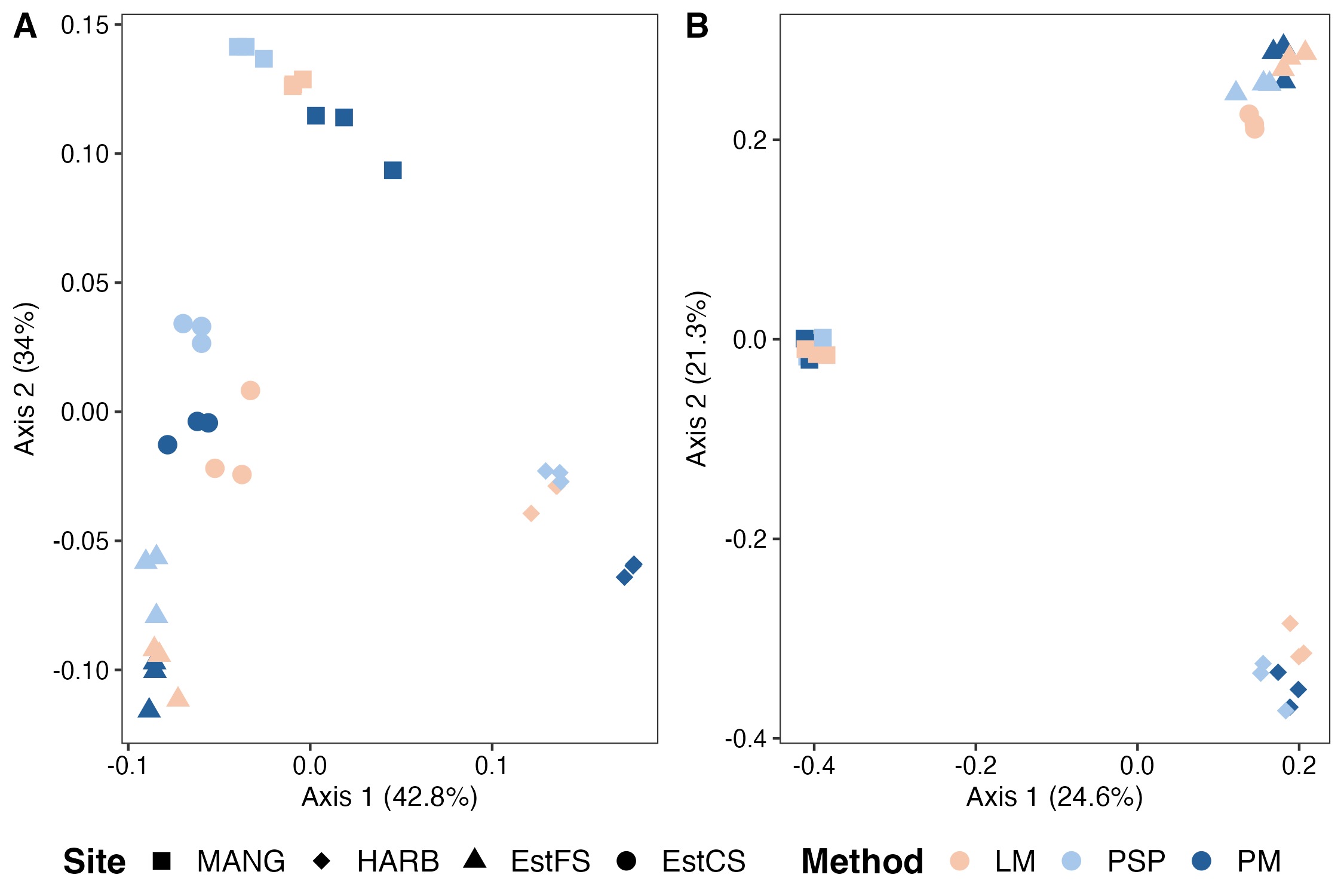

Supplement: S3 Fig — (A) PCoA on weighted UniFrac distances of prokaryotic taxa. (B) PCoA on unweighted UniFrac distances of eukaryotic taxa. LM: Laboratory-made method. PSP: PowerSoil Pro. PM: PowerMax Soil. (TIFF) [file pone.0343743.s005.tiff]

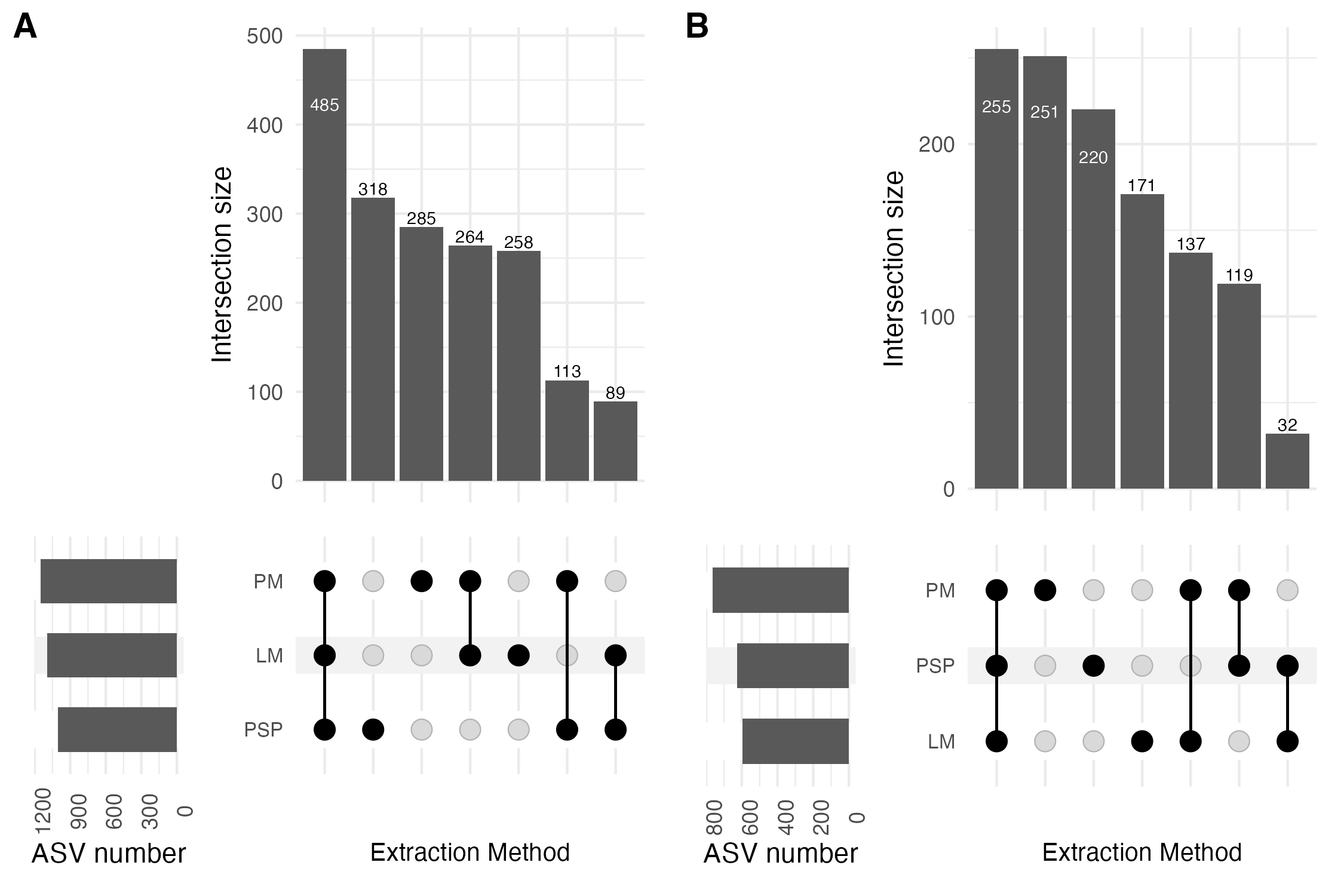

Supplement: S4 Fig — (A) Prokaryotic ASVs. (B) Eukaryotic ASVs. Shared and unique ASVs were defined based on presence in all three replicates of a given sediment for each method. (TIFF) [file pone.0343743.s006.tiff]
