# Supplementary material for: Cost-effective DNA extraction method optimized for high yield and long fragments from coastal sediments
Source: PLoS One. 2026 Feb 24;21(2):e0343743. doi: 10.1371/journal.pone.0343743 (PMC12931794; doi:10.1371/journal.pone.0343743)
Supplement: Attachment — Submitted filename: Response to Reviewers.docx [file pone.0343743.s009.docx]

Dr. Raffaella Casotti

*Academic Editor*

PLOS One

December 18, 2025

Dear Dr. Casotti,

Thank you for giving us the opportunity to submit a revised copy of the manuscript entitled “Cost-effective DNA extraction method optimized for high yield and long fragments from coastal sediments”. We appreciate the time that you and the reviewers dedicated to providing constructive feedback on our work. Suggestions made were incorporated in the Manuscript with tracked changes and discussed below. All page numbers refer to the revised manuscript file with tracked changes.

# Response concerning the journal requirements:

1. Please ensure that your manuscript meets PLOS ONE's style requirements, including those for file naming. The PLOS ONE style templates can be found at <https://journals.plos.org/plosone/s/file?id=wjVg/PLOSOne_formatting_sample_main_body.pdf> And <https://journals.plos.org/plosone/s/file?id=ba62/PLOSOne_formatting_sample_title_authors_affiliations.pdf>

Author response: We checked style formatting once again to make sure the manuscript meets PLOS ONE’s requirements.

2. In your Methods section, please provide additional information regarding the permits you obtained for the work. Please ensure you have included the full name of the authority that approved the field site access and, if no permits were required, a brief statement explaining why.

Author response: We added the information we judged necessary about site access:

“While no permits were required to access the publicly accessible sampling sites in France, sampling in Gabon was conducted under authorization from the Centre National de la Recherche Scientifique et Technologique (CENAREST).” Line 78.

3. We note that you have included the phrase “data not shown” in your manuscript. Unfortunately, this does not meet our data sharing requirements. PLOS does not permit references to inaccessible data. We require that authors provide all relevant data within the paper, Supporting Information files, or in an acceptable, public repository. Please add a citation to support this phrase or upload the data that corresponds with these findings to a stable repository (such as Figshare or Dryad) and provide and URLs, DOIs, or accession numbers that may be used to access these data. Or, if the data are not a core part of the research being presented in your study, we ask that you remove the phrase that refers to these data.

Author response: We understand the issue raised. This term was misused, the data associated with this sentence is in fact already presented in the text (DNA concentrations in MANG and HARB for each protocol). Therefore, we removed “data not shown” from the manuscript, line 263.

4. PLOS ONE now requires that authors provide the original uncropped and unadjusted images underlying all blot or gel results reported in a submission’s figures or Supporting Information files. This policy and the journal’s other requirements for blot/gel reporting and figure preparation are described in detail at <https://journals.plos.org/plosone/s/figures#loc-blot-and-gel-reporting-requirements> and <https://journals.plos.org/plosone/s/figures#loc-preparing-figures-from-image-files> When you submit your revised manuscript, please ensure that your figures adhere fully to these guidelines and provide the original underlying images for all blot or gel data reported in your submission. See the following link for instructions on providing the original image data: <https://journals.plos.org/plosone/s/figures#loc-original-images-for-blots-and-gels>

Author response: We added the original uncropped gel results present in S1 File. We included the file “S1_raw_images.tif” in the submission, in compliance with PLOS ONE requirements.

# Responses to comments and questions of Reviewer 1:

1. The authors present a laboratory-made (LM) DNA extraction method benchmarked against two commercial kits for coastal sediments. The LM approach produced substantially higher yields and longer DNA fragments, enabling improved recovery of eukaryotic diversity. Although more labor-intensive, it proved reproducible, cost-effective, and well-suited for large-scale or resource-limited studies. Overall, the paper makes a valuable contribution, but certain sections (methods clarification, conceptual definitions, and expanded comparisons) should be improved for clarity and completeness.
   Abstract
   The sentence “While prokaryotic diversity metrics were largely similar across methods, we observed differences in eukaryotic richness that were likely influenced by the amount of sediment processed” could be worth keeping, but you should clarify why this observation is relevant for the paper’s scope. Does it support the strength of your method (better for eukaryotes)? Or does it show a limitation (sensitive to sample size)? At present, it feels underdeveloped.

Author response: Thank you for your comment, we meant it as a limitation of the method and the importance of sample size for the study of microeukaryotes. We thus changed the sentence for:

“While prokaryotic diversity metrics were largely similar across methods, we observed differences in eukaryotic richness that were likely influenced by the amount of sediment processed. This demonstrates the higher importance of sample amount when assessing microeukaryotic diversity.” Line 35.

2. Line 49: The sentence “However, those technologies are still dependent on DNA extraction methods” — but isn’t this true for other sequencing or analysis approaches as well? Please clarify the distinction you are making here, otherwise the statement sounds too general.

Author response: Thank you for your suggestion. We agree that the statement is indeed too general. The goal was to indicate the importance of DNA extraction as it is the first step before studying taxonomy using molecular tools. To clarify the sentence, we improved it as such:

“However, those technologies are still dependent on DNA extraction methods, indicating the importance of the efficiency of DNA extractions to recover high yields of high-quality DNA (long fragments and inhibitor free) and capture broader microbial diversity.” Line 62.

3. Line 55: Since this is a generalist journal, please briefly define alpha and beta diversity for non-specialist readers (one short clause each should be enough).

Author response: Thank you for your comment. We added a definition of alpha diversity “within-sample taxonomic richness and evenness” and beta diversity “between-sample compositional dissimilarity”. Line 71.

4. Please expand slightly on the difference between NGS shotgun sequencing and metabarcoding (at least two sentences). For example: emphasize that metabarcoding targets specific loci (e.g., 16S, COI), which can bias taxonomic resolution, whereas shotgun metagenomics enables recovery of longer fragments and broader taxonomic/functional profiles.

Author response: Thank you for your comment. We have expanded the description of the differences between metabarcoding and shotgun metagenomics in the revised manuscript:

“Metabarcoding targets specific genetic loci (e.g., fragments of 16S or 18S rRNA genes) to generate taxonomic profiles from established marker genes with high coverage of the genetic diversity for the targeted loci. In contrast, shotgun metagenomics sequences all DNA present in a sample, providing access to wider range of taxonomic and functional information, while providing less sequencing depth on each sequenced genomic region. Third-generation sequencing further improves shotgun metagenomics by producing long, continuous reads that help resolve complex regions and strengthen genome reconstruction. It also enables long-read metabarcoding, allowing full-length marker sequencing and improved taxonomic resolution.” Line 55-61

5. Line 61: Please describe the sediment type in more detail (grain size, organic content, coastal location, depth collected, etc.). This information is important to contextualize yield and inhibition challenges.

Author response: Thank you for this suggestion. To improve clarity for readers, we added the note “characteristics, location, organic and water content are described in Table 1” (Line 78). We believe the manuscript already includes key contextual information, namely:

(1) Coastal location: “in a coastal area (near Alembétogo, Gabon)” Line 82; “a small marina (Oursinieres harbor, Le Pradet, France)” Line 84; “a subterranean estuary located on Pellegrin Beach (La Londe-les-Maures, France)” Line 85.

(2) Sampling depth: “depth of 5 centimeters.” Line 82; “~ 0.2-meter depth” Line 87.

We trust directing readers to Table 1 for sediment characteristics, organic content, and water content provides the additional details you requested.

6. Line 77: Clarify what exactly was performed in triplicate (DNA extractions? PCRs? sequencing libraries?). Also, explain why triplicates were necessary (e.g., to ensure reproducibility, to capture variability within sediment aliquots).

Author response: Thank you for pointing this out. We added the sentence: “At each site, sediments were collected in three independent 50 mL Corning tubes to capture within-site variability.” (Line 89) and modified the sentence explaining how DNA extractions were performed “For each DNA extraction method, three separate DNA extractions were performed on three thawed environmental triplicates for each sediment.” Line 99.

7. Line 93: The statement “protocol should be adapted” is vague. If you mean that increasing input material often leads to inhibitor co-extraction, please state that explicitly and explain what adaptations you recommend or tested.

Author response: Thank you for your comment. We agree with the fact that increasing the starting mass increases the co-extraction of inhibitors. Therefore, we clarified the sentence: “To recover more DNA, LM can be adapted for larger amounts of starting material. To prevent increased co-extraction of inhibitors, lysis and precipitation volumes must be increased proportionally to maintain the same sediment-to-reagent ratio. For example, successful extractions were performed using 2 g of sediment in 6.6 mL of lysis buffer and CTAB in 15 mL Corning tubes.” Line 117.

8. Line 175: “Clenned?” — please check this typo. If you mean cleaned libraries, specify the parameters: bead-to-sample ratios, fragment size ranges retained, or kits used. This will improve reproducibility.

Author response: Thank you for your comment. Eurofins Genomics provided us – in their terminology – “cleaned” reads that were pre-processed using Cutadapt software. To clarify, we modified the two sentences as such: “Eurofins Genomics provided MiSeq reads that were trimmed of Illumina adaptors using Cutadapt software v2.7 [23]. Reads of 16S and 18S fragments were then separately processed […]” Line 211.

9. Line 200: The authors already mention “Kruskal–Wallis p-value cutoff of 0.05” (line 195). Later (line 200), you repeat “p-value threshold for statistical significance was 0.05”. Consider consolidating into one clear statement at the end.

Author response: Thank you for pointing this out. The first occurrence of this sentence was to describe the parameters used to perform the LEfSe analysis, and the second occurrence to generalize to other statistical tests. To improve intelligibility, we modified the first occurrence: “LEfSe parameters were the following: norm = “CPM”, taxa_rank = “none”, kw_cutoff = 0.05, lda_cutoff = 2.” Line 236.

10. Line 211: When mentioning statistically significant or nt, include p-val and test.

Author response: Thank you for pointing this out. We added the test and the p-value when we mentioned “significant” and either information was missing.

11. Consider adding a brief statement about limitations (e.g., only sandy sediments tested, other sediment types may behave differently).

Author response: Thank you for your comment. We added a brief statement in the Discussion to highlight the fact that results vary between muddy and sandy sediments we tested:

“Our results show that DNA extraction yields and fragment sizes vary between muddy and sandy sediments. For muddy sediments, no general trend between methods indicates that the efficiency of methods was sediment specific. For sandy sediments, the LM method outperforms both commercial kits in terms of DNA extraction yield up to a factor of 13.” Line 519.

# Responses to comments and questions of Reviewer 2:

The paper « Cost-effective DNA extraction method optimized for high yield and long fragments from

coastal sediments » proposes a new « home-made » DNA extraction method efficient for sediments, particarly for sandy or low-biomass sediments. Moreover, this method could be very useful for studies requiring high-quality DNA, specifically for long-fragments analysis, like metabarcoding or metagenomics using third generation sequencing technologies.

This protocol was compared to very widespread commercial kits used in genomic environmental studies, and different soil compositions were tested, which was very important for the protocol validation. Moreover, DNA extractions were realized in triplicate for each method to check the repetability and reproductibility of the lab work.

To check the efficiency of the method, 3 parameters have been checked : DNA extraction yield, DNA fragment size, and alpha and beta biodiversity based on 16S (prokaryotes) and 18S (eukaryotes) markers.

I really appreciated the details in the protocol, which can be directly applied in the lab. The references and the costs are clearly listed.

Moreover the methods used to measure the quantities and the qualities of DNA extracts are really precises and showed in the paper.

Nevertheless, I found the protocol quite time-consuming and requires long reagent preparation time steps. These 2 factors have to be considered when you have a lot a samples to process.

I also have some questions an remarks about the strategy of study validation :

1. Did you try to modify some parameters from commercial kits PSP and PM ? I know lysis steps can be very rough. Did you test different lysis condition sto to optimize the integrity of DNA extracts?

Author response: Thank you for your comment. We did not modify lysis steps of the commercial PSP and PM kits. Reducing mechanical lysis would likely decrease DNA yield rather than improve fragment size (e.g., Ma et al. 2020, https://doi.org/10.3389/fmicb.2020.581227). Although such optimization could be explored in future work, our objective here was to develop a cost-effective and efficient alternative to commercial kits, particularly one suitable for use in developing countries.

2. I am quite sceptical regarding eukaryotes PCR amplification step :

2.a. Go Taq polymerase (Promega) is known to not be the best choice to amplify marine microbes, specifically protists. Most of protists taxa have high-rich GC content, so Kapa and Phusion polymerases are preferably used.

5. To conclude, I would suggest to do more amplification tests on V7 marker and longer fragments (like entire 18S marker or more) and using a different Taq such as Phusion High Fidelity F532L to prove the efficiency of the method.

Author response: Thank you for this constructive suggestion. We agree that polymerase choice can influence amplification success. Following your recommendation, we tested an alternative high-fidelity enzyme (Phusion High Fidelity Master Mix with GC Buffer, F532) with the same starting DNA amount and adjusted denaturation temperatures according to the manufacturer’s guidelines. However, this polymerase did not improve amplification success: samples that failed with GoTaq also failed with Phusion (Sediment PelCS extracted with PSP), and overall amplification rates across all samples were lower with Phusion than with GoTaq (19 vs. 33 positive amplifications out of 36 samples, respectively). These results suggest that amplification failures were not primarily driven by polymerase choice, but likely by sample-specific factors such as DNA integrity or inhibitor carry-over.

To document this comparison, we provide below the gel image showing amplification results obtained with the Phusion polymerase. For this electrophoresis, 5 µL of PCR product were loaded per well; migration was performed at 135V for 20min, using the FastGene 100bp DNA Marker (Nippon Genetics).

2.b. Could you explain the choie of V7 marker and primers please ? I totaly approve V7 is a good candidate regarding sedaDNA metabarcoding studies, but n most of modern marine eukaryotic metabarcoding studies (coastal and open ocean areas), V4 or V9 markers are more used. (ie Zimmermann et al. 2024 DOI:10.1002/edn3.580, Mahe et al. 2017 DOI:10.1038/s41559-017-0091).

Author response: Thank you for that interesting comment. As noted in Zimmermann et al. (2024), the V4 region generally provides higher taxonomic resolution than the V7 region. However, the V7 region remains a suitable marker in terms of broad taxonomic coverage – often outperforming the V9 region – and continues to be used in recent marine studies (e.g., https://doi.org/10.1016/j.funeco.2025.101460). Using shorter markers such as V7 or V9 can also help mitigate amplification challenges, particularly in low-biomass environments where longer fragments (e.g., V4) amplification may fail more frequently. Importantly, Zimmermann et al. (2024) was not yet available at the time we designed our study, but we plan to consider the V4 region in future work. While the choice of 18S markers can be discussed at length, we do not expect that using the V4 region in this study would have altered the conclusions regarding the performance of the proposed method.

3. How many replicates did you do for each of your DNA amplifications ? I didn’t find the information in the text.

Author response: Thank you for your question. Biological replicates were extracted separately to ensure reproducibility. Each DNA sample was then amplified once (i.e., no technical replicates was performed for the PCR amplification). To clarify the Methods section, we added information about PCR replicates in the “PCR amplification and sequencing” section: “Each DNA sample was amplified once; no technical PCR replicates were performed.” Line 199. We also clarified in the “Sediment collection” which procedures were performed in triplicates (Lines 89-91 and 99).

4. You highlight the potential of the LM method to recover long-fragments of DNA. Why didn’t you check alpha and beta diversity using 3rd generation sequencing like PacBio or ONT ? (ie https://doi.org/10.1101/2025.07.20.665787, https://doi.org/10.3897/mbmg.9.163750 )

Author response: Thank you for your insightful comment. Indeed, third-generation sequencing technologies such as PacBio or ONT offer higher taxonomic resolution, which would enable more accurate comparisons of method-specific taxa. However, for basic diversity metrics, Bludeau et al. (2024) showed that metabarcoding using shorter markers than V7 (V9 in their case) yields beta-diversity patterns comparable to those obtained from full-length 18S rRNA sequencing with ONT (https://doi.org/10.1002/edn3.70084). Additionally, the implementation of long-read sequencing was not feasible within the resource constraints of this project.

# Responses to comments and questions of Reviewer 3:

This manuscript presents a laboratory-made (LM) DNA extraction method optimized for high yield and long DNA fragments from coastal sediments, comparing its performance against two commercial Qiagen kits (PowerSoil Pro and PowerMax Soil). The study addresses a relevant methodological bottleneck in environmental DNA research, extracting high-quality DNA from complex, low-biomass matrices such as sandy or organic-rich sediments, with the goal of providing an affordable, non-hazardous alternative suitable for large-scale or resource-limited settings.

The topic is scientifically relevant and of clear practical value. The study is generally well structured and clearly written. However, some aspects of the methodological comparability, data normalization, and interpretative depth limit the robustness of the conclusions. While the LM method appears promising, certain parts of the work require clarification, additional controls, and normalization to ensure fair comparisons among extraction approaches.

1. How many independent replicates were performed for each extraction protocol and sediment type? Please specify whether biological and technical replicates were included. This is essential to assess reproducibility and to validate the statistical comparisons (ANOVA, PERMANOVA).

Author response: Thank you for pointing this out. We added the sentence: “At each site, sediments were collected in three independent 50 mL Corning tubes to capture within-site variability.” (Line 89) and modified the sentence explaining how DNA extractions were performed “For each DNA extraction method, three separate DNA extractions were performed on three thawed environmental triplicates for each sediment.” Line 99.

2. No extraction blanks or negative controls appear to have been sequenced. These are essential for detecting contamination, especially in low-biomass matrices.

Author response: Thank you for highlighting the importance of sequencing negative controls. In our study, extraction blanks were systematically included for all extraction methods. For PSP and PM, extraction blanks did not yield any amplification. For LM, a faint amplification was observed, but the amplicon concentration remained below the threshold required by the sequencing company for library preparation and sequencing. As a result, no sequencing data could be generated from these blanks. We acknowledge the limitations that this implies for contamination detection, but since samples DNA extracts yielded strong amplifications the risk of contaminating sequences appears very low. This point is now clarified in the manuscript:

“For LM, a faint amplification was observed in the extraction blanks; however, the DNA quantity was insufficient for library preparation and sequencing. Consequently, no sequencing data could be obtained from the LM extraction blanks.” Line 202.

3. The authors appropriately normalized DNA yields to both sediment dry weight (µg DNA g⁻¹) and total elution volume, as stated in the Methods (lines 220–222). This normalization ensures that reported extraction efficiencies are mathematically comparable across the three methods (LM, PSP, and PM). However, even though yield normalization was properly performed, the biological and ecological comparability among extraction protocols remains partially limited due to the large differences in input sediment mass and lysis chemistry: LM and PSP processed 200–250 mg of sediment, whereas PM used 5 g, potentially capturing a broader microbial and eukaryotic diversity simply by integrating more microhabitats. The LM protocol relies on enzymatic and detergent-based lysis (CTAB/SDS + Proteinase K), while PSP and PM use mechanical bead-beating, which can favor the extraction of different cell types (e.g., Gram-positive bacteria, spores). These factors could influence both DNA yield and community composition, independent of extraction efficiency. Therefore, the manuscript should explicitly acknowledge in the Discussion that, while yield data were normalized correctly, the ecological representativeness of each extraction remains partly influenced by input mass and lysis mechanism. To strengthen this comparison, I recommend including a PCoA or NMDS plot showing beta-diversity differences between extraction protocols across sediment types. This would visually confirm whether extraction method drives distinct community structures.

Author response: Thank you for your comment. We agree that differences in input sediment mass and lysis chemistry can influence both DNA yield and community composition independently of extraction efficiency. These limitations were acknowledged in our Discussion section (lines 550-558), where we note that the LM, PSP, and PM protocols differ in processed sediment mass, and such methodological differences may affect ecological representativeness and the taxa recovered. We further discuss the influence of lysis chemistry on extraction outcomes in lines 571-573. To improve clarity, we added a sentence in the Discussion about lysis chemistry and input mass (Line 548).

Following your recommendation, we have now added two PCoA plots based on beta-diversity metrics to the Supplementary Material (S3 Fig). This figure illustrates the community-level differences associated with each extraction method across sediment types. The manuscript has been updated to explicitly refer to this supplementary figure in the Results section: “Although the choice of extraction protocol influenced community structure within each sediment type, overall community composition remained clearly differentiated between sediment types regardless of the protocol used (see PCoAs in S3 Fig).” Line 367.

4. It is unclear whether sequencing depth normalization or rarefaction was performed before comparing alpha and beta diversity. This is critical for metabarcoding data to ensure comparability across methods and sediments, The Methods should specify how read counts were normalized before ANOVA/PERMANOVA tests (e.g., rarefaction to minimum library size, relative abundance transformation, or DESeq2 variance stabilization). To complement this, an Upset plot illustrating shared vs unique ASVs among the different extraction protocols would provide a more intuitive visualization of overlap and method-specific recovery of taxa. This approach would highlight whether the LM method captures a unique subset of taxa or primarily overlaps with commercial kits.

Author response: Thank you for pointing this out. We had omitted information on how sequencing data were normalized. We have therefore added the following sentence “Read counts were normalized to relative abundances using Total Sum Scaling (TSS).” (Line 227).

Following your recommendation, we also added an Upset plot in the Supplementary Material (S4 Fig) to illustrate shared and method-specific ASVs across extraction protocols. This analysis shows that, for both prokaryotic and eukaryotic communities, a large fraction of ASVs was shared among the three methods. Each protocol also recovered a notable number of unique ASVs, and the LM method exhibited the strongest overlap with the PM kit.

The Methods section has been updated accordingly (Lines 236–240), and the corresponding results are now presented in Lines 447–451 and 494–498. The Discussion has also been amended to reflect these findings:

“Despite differences in extraction methods, beta diversity analysis showed that sediments were clustered by site regardless of the extraction method, with most taxa being shared across methods. Nonetheless, each method recovered a substantial number of unique ASVs in both prokaryotic and eukaryotic datasets, likely reflecting differences in lysis chemistry and input sediment masses.” Lines 546–550.

Apart from the Reviewers comment, we would also like to bring your attention to three errors we identified:

- A grammar error line 359: “Beta diversity measurements **is**” replaced by “Beta diversity measurements **are**”
- EDTA concentration: “100 mM” replaced by “10 mM”. This has been corrected in the manuscript (line 125) and in S1 File.
- Affiliation information concerning the author Eléonord Mayissah Moungues. EMM is affiliated to both Université de Toulon (France) and the LARME (Gabon) (line 9)

We hope you find our revised manuscript suitable for publication and look forward to hearing from you in due course.

Sincerely,

Elisabeth Navarro

Toulon University, Mediterranean Institute of Oceanography

Avenue de l’Université, 83130 La Garde, FRANCE

Tel.: +33 781 924 666

Email: elisabeth.navarro@ird.fr
