# Supplementary material for: Cost-effective DNA extraction method optimized for high yield and long fragments from coastal sediments
Source: PLoS One. 2026 Feb 24;21(2):e0343743. doi: 10.1371/journal.pone.0343743 (PMC12931794; doi:10.1371/journal.pone.0343743)
Supplement: Attachment — Submitted filename: Response_to_Reviewers_auresp_2.docx [file pone.0343743.s010.docx]

Dr. Raffaella Casotti

*Academic Editor*

PLOS One

February 6, 2026

Dear Dr. Casotti,

Thank you for giving us the opportunity to submit a revised copy of the manuscript entitled “Cost-effective DNA extraction method optimized for high yield and long fragments from coastal sediments”. We appreciate the time that you and the reviewers dedicated to providing constructive feedback on our work. Suggestions and comments made were discussed below.

# 5. Is the manuscript presented in an intelligible fashion and written in standard English?

# PLOS ONE does not copyedit accepted manuscripts, so the language in submitted articles must be clear, correct, and unambiguous. Any typographical or grammatical errors should be corrected at revision, so please note any specific errors here.

# Reviewer #2: No

# Author response: Reviewer #2 initially indicated that the manuscript was presented in an intelligible manner and written in standard English. We were therefore somewhat surprised that language-related concerns were raised in the second round of review, as only minor modifications were made to the manuscript. Nevertheless, we have carefully revised the text to further improve clarity and readability throughout.

# 6. Review comments to the Author (Reviewer #2)

Comments on 2a and 5:

Many thanks for your tests. But it is still not clear. I suggested in my review to do some amplifcation tests on 18S-V7, but in your new submission, you studied both 18S-V7 (eukaryotes) and 16S-V4V5 (prokaryotes). Which marker do you finally amplified with the Phusion ? 18S-V7 or 16S-V4V5 ?

Author response: Thank you for your comment and we apologize for the lack of clarity. In the original manuscript, both 18S-V7 and 16S-V4V5 were amplified using the Promega polymerase. Following your recommendation during the first round of review, we tested the 18S-V7 marker with the Phusion polymerase, as Promega may not be optimal for marine microbes, especially protists. We apologize for not clearly stating that these amplification tests were performed only on the 18S-V7 marker. These tests resulted in a lower amplification success with Phusion compared to Promega. As a result, we retained the Promega polymerase for all amplifications presented in the manuscript, for both 18S-V7 and 16S-V4V5.

Comments on 3:

Thank you very much for your answer. I am disappointed to not have technical replicates in the PCR. We know that amplification step adds a lot of biaises for metabarcoding studies, even at 25 cycles, that’s why technical replicates (at least 3) are recommended, specially in sediments. Maybe you had to face resource constraints, but it is a critical point.

Author response: Thank you for your comment. We agree that PCR technical replicates are recommended in metabarcoding studies to limit amplification biases. In this study, we prioritized experimental replicates, which we consider to capture a broader source of variability than PCR-induced errors alone. We acknowledged this limitation in the manuscript (line 190) and will implement PCR technical replicates in future work.

Comments on 4:

Thank you very much for your explanations and I totally understand your resource constraints. Nevertheless, this LM DNA extraction protocol is promoted for getting high-molecular-weight DNA. It would have been interesting to show some results on ONT or PacBio.

Author response: Thank you for your comment. While we acknowledge that ONT or PacBio sequencing can provide higher taxonomic resolution, the primary aim of this study was not to optimize taxonomic precision but to compare community-level patterns and method-specific differences across extraction protocols. In this context, previous work has shown that alpha-diversity patterns are consistent between short- and long-read metabarcoding approaches (Mittelstrass et al. 2025, <https://doi.org/10.1186/s40793-025-00712-7> ; Doorenspleet et al. 2025 <https://doi.org/10.7717/peerj.19158>). Similar results were found comparing beta-diversity patterns (Doorenspleet et al. 2025; Bludeau et al. 2024 <https://doi.org/10.1002/edn3.70084>). Consequently, we do not expect the use of long-read sequencing to substantially alter the main conclusions of this study, and we therefore consider the current approach appropriate to address our research objectives.

I still have new questions about the modified article :

Line180-182 for 18S-V7 failed amplifications, you mentionned you reamplifed with 20 ng of DNA.

Did you test with the PCR with less DNA amounts (1 or 0.1 ng startin material) ? Despite the fact DNA extraction methods have a lot of purification steps, there are still a lot of inhibitors in DNA extracts from sediments. If you dilute the DNA input in the PCR, you also dilute PCR inhibitors and increase the amplification yield.

Author response: Thank you for raising this point. We agree that diluting DNA input can in some cases improve PCR amplification by reducing the concentration of inhibitors, particularly in sediment-derived DNA extracts. In this study, samples that failed to amplify with the 18S-V7 marker successfully amplified with the 16S-V4V5 marker, suggesting that PCR inhibition was unlikely. Instead, these failures were more consistent with a low amount of target DNA, possibly related to the low eukaryotic biomass commonly observed in sediment samples. This interpretation is supported by the improved amplification success of the 18S-V7 marker using more input DNA (20 ng instead of 10 ng). In addition, preliminary tests performed on similar sediment types using lower DNA inputs (1 ng) did not improve amplification success for the 18S-V7 marker. These results suggest that, in our case, amplification failure was more likely related to low target DNA abundance rather than PCR inhibition.

S2_File : it is not « furnisher », but « Supplier ». And Fisher brand is the same than Thermofisher.

Author response: Thank you for this correction. We replaced “Furnisher” with “Supplier” and corrected “Fisher” for “ThermoFisher” in S2_File.

We hope that our responses have satisfactorily addressed the points raised and that our manuscript will be considered for publication.

Sincerely,

Elisabeth Navarro

Toulon University, Mediterranean Institute of Oceanography

Avenue de l’Université, 83130 La Garde, FRANCE

Tel.: +33 781 924 666

Email: elisabeth.navarro@ird.fr
